# Prevalence of overweight/obesity, anaemia and their associations among female university students in Dubai, United Arab Emirates: a cross-sectional study

**DOI:** 10.1017/jns.2020.23

**Published:** 2020-07-06

**Authors:** Haleama Al Sabbah

**Affiliations:** Public Health Nutrition Department, Zayed University, Dubai, United Arab Emirates

**Keywords:** Anaemia, Females, Obesity, Overweight, United Arab Emirates, UAE, United Arab Emirates, WC, waist circumference

## Abstract

The present study assessed the associations of overweight, obesity and anaemia with selected lifestyle factors, total body fat and abdominal obesity among female university students in Dubai. A total of 251 female students from a national university in Dubai participated in the present study. Weight, height, waist circumference, Hb level and total body fat percentage were measured. Participants also completed a self-reported questionnaire that included items related to the factors of obesity, anaemia and lifestyle. The study was approved by the University Ethical Committee. Almost one-third of the participants were overweight/obese; 8⋅5 % had abdominal obesity while 18⋅1 % had anaemia. Out of the total, 71⋅7 % reported that they have irregular meals and the highest percentages were found among obese (89⋅3 %) and overweight (78⋅0 %) compared with normal-weight (65⋅4 %) students (*P* < 0⋅05). Overweight/obese students reported that they exercise more than those of normal weight (*P* = 0⋅05). Students with anaemia reported less exercise than students without anaemia (*P* = 0⋅05). Also, the percentage of total body fat was found to be the highest (38⋅9 %) among students with anaemia (*P* < 0⋅05). Overweight, obesity and anaemia are prevalent among female university students. Anaemia seems to be associated with the percentage of total body fat, lack of physical activity and junk food. Further studies are required to investigate the detailed dietary habits of overweight and obese young adult females with anaemia.

Obesity and overweight are major public health problems that are associated with a higher mortality rate worldwide^(1)^. Obesity is a medical condition in which excess body fat has accumulated to the extent that it may have an adverse effect on health, leading to reduced life expectancy and/or increased health problems^(2)^. Previous studies have shown that it is more prevalent among women than men and that young adult females in general are at an increased risk of developing anaemia^(2–4)^.

A decreased count of erythrocytes and diminished Hb levels are often what characterise anaemia^(3)^. The occurrence of anaemia is enhanced by factors including puberty, excessive menstrual losses, giving birth, poor dietary choices and insufficient Fe intake from animal-based foods^(4)^. Anaemia has been linked to several health consequences such as impaired physical and cognitive performance, and increased risk of maternal and child mortality^(3,5)^. Food habits can result in anaemia and increase the risk of obesity, particularly among young females^(6–10)^. Factors involved include skipping breakfast, reduced intake of fruits and vegetables and increased consumption of sugary drinks and high-energy foods^(11,12)^. Several previous studies indicated that anaemia is more prevalent among overweight and obese adults in general^(8,9,13,14)^, and also among overweight children, adolescents and young adults^(8,15,16)^.

A study in the Kingdom of Saudi Arabia showed that the prevalence of anaemia among female university students was 23⋅9 %^(17)^. Given the health consequences of anaemia, its increased prevalence among females and the obesity epidemic worldwide, studying the prevalence of obesity and overweight and their associations with Hb level and food habits is a major part of the present study to guide future studies and interventions.

International and regional studies show an increase in obesity rates and highlight that factors associated with it are multi-factorial particularly among university students, but there has been little relevant research relating anaemia to obesity and total body fat in the United Arab Emirates (UAE)^(1,2,15–17)^.

There are no data available at all in the literature from the UAE. Lack of studies from the UAE population makes the present study very important to be as a reference and base for researchers and decision makers. The present study will add new knowledge to the international studies about Emirati people living in the UAE. Moreover, the present study will add estimates from the Global Burden of Disease Study and other researchers and decision makers might use this data to design later national data and other studies. The main objective of the present study is to assess the prevalence of overweight, obesity, anaemia and their associations with the eating habits, physical activity and total body fat among female students at one of the national universities in the UAE.

## Methods

### Study design

A cross-sectional study was conducted on 251 female students at one of the national universities in the UAE from 24 November to 10 December 2014. The present study was conducted according to the guidelines laid down in the Declaration of Helsinki and all procedures involving human subjects were approved by Zayed University Ethical Committee (no. ZU14_052_F). Written informed consent was obtained from all subjects. Students were informed that participation in the study is voluntary, and before data collection students were provided with full information about the study and given the opportunity for potential participants to ask any question before they signed the written informed consent.

### Population and sampling

The total number of female students enrolled at the university in autumn 2014 at the Dubai campus was approximately 3000 Emirati undergraduate students. In order to have a 10 % of the total population representative sample from all colleges, the estimated sample size was 300 students selected by using a systematic random sampling from: Colleges of Arts, Business, Communication and Media, Education, Sustainability and Human Sciences, Technology, and University College. Students' names and numbers were imported from the university's Banner Web in the 2014 autumn semester. An interval of 10 was used to select students randomly from the imported list and a random starting number was obtained by using Excel. In addition, the Students' Affairs Office at the university and all university teachers were informed about the present study in order to encourage students to participate. Since they were blinded from knowing who was selected, they advertised the study to all their students.

The sample size calculation was based on the formula of infinite population sample size (SS) equation: SS = (*z*^2^ x *p* x *q*)/*d*^2^; where *z* is the α risk expressed in the *z*-score, *p* is the predicted prevalence, *q* is (1 – *p*) and *d* is the absolute precision. In the present study, a CI of 95 % (α = 0⋅05), hence a *z*-score of 1⋅96, a predicted prevalence of anaemia of *p* = 0⋅20 and an absolute precision of *d* = 0⋅05 were used. Thus, a sample of 246 students was required.

### Data collection

A total of 307 randomly selected students were invited via emails to come to the University Nutrition Laboratory and participate in completing the study questionnaire and to measure their weight, height, waist circumference (WC), total body fat and Hb level. One female nurse and two research assistants were recruited and trained to take the measurements for the participating students. In total, 251 students came to the laboratory and agreed to participate in the present study, resulting in a response rate of 81⋅8 %. Two senior students on their final year from the Department of Public Health Nutrition who received training on nutrition education under the supervision of a professor in nutrition were giving nutrition education to students who had low Hb level and students with obesity or underweight. In addition, students with a low Hb level were referred to the university health centre for follow-up.

### Procedures and measurements

Participants completed a self-reporting questionnaire that included items related to obesity, anaemia and lifestyle. The questionnaire was developed based upon information existing in the related literature and validated by a professor in nutrition then piloted on twenty students who are not included in the present study analysis.

Weight and height were measured by using calibrated scales and stadiometers, WC was measured under clothing by flexible measuring tapes. Height and WC were measured to the nearest 0⋅5 cm. Body weight was measured to the nearest 0⋅1 kg. The participants were weighed with a minimum of clothing and no shoes. BMI was calculated by dividing weight in kg by the height in metres squared (kg/m^2^). BMI was categorised into underweight (BMI<18⋅5 kg/m^2^), normal weight (BMI = 18⋅5–24⋅9 kg/m^2^), overweight (BMI = 25–29⋅9 kg/m^2^) and obese (BMI ≥30 kg/m^2^)^(2)^. Abdominal obesity was assessed using the WC measurement and was based upon the WHO criteria for cut-offs (WC ≥88 cm for females and ≥102 cm for males)^(2)^. Total body fat percentage was measured by a Tanita machine at the university nutrition laboratory.

Hb level was measured by using a Hemocue analyser. Blood samples were collected by a professional nurse from participating students using finger pricks. According to the WHO, the level of anaemia among non-pregnant women aged 15 years old and above was defined by Hb level below 120 mg/l^(18)^.

### Statistical analyses

Data obtained were analysed using the SPSS statistical package (IBM). The χ^2^ test was used to compare frequencies between categorical variables. The significance level was set at 0⋅05.

## Results

Table 1 shows the sample's sociodemographic characteristics. The mean age was 23⋅1 (sd 5⋅3) years. The majority perceived having a moderate economic status (82⋅3 %). Of the total, 7⋅6 % reported being married and 4 % were engaged.

**Table 1.** Sociodemographic characteristics (*n* 251) (Numbers of subjects and percentages)

	*n*	%
Age		
≤20 years	143	57⋅7
>20 years	105	42⋅3
Academic level		
1st year	40	15⋅9
2nd year	47	18⋅7
3rd year	73	29⋅1
4th year	91	36⋅3
Economic status perception		
High	44	17⋅7
Moderate	204	82⋅3
Marital status		
Single	221	88⋅4
Engaged	10	4⋅0
Married	19	7⋅6

The prevalence of overweight and obesity was found to be 29⋅3 % (17⋅4 and 11⋅9 %, respectively). The prevalence of abdominal obesity (WC ≥88 cm) was 8⋅5 %, with a mean WC of 71⋅6 (sd 10⋅5) cm. The prevalence of anaemia was 18⋅1 %, with a mean Hb level of 129⋅5 (sd 13) mg/l, ranging between 86 and 161 mg/l (Table 2).

**Table 2.** Prevalence of overweight, obesity and anaemia (Numbers of subjects and percentages, ranges, and mean values and standard deviations)

Variables	*n*	%	Range	Mean	sd
Measured BMI (kg/m^2^)			14⋅2–43⋅7	23⋅1	5⋅3
Underweight	37	17⋅0			
Normal weight	117	53⋅7			
Overweight	38	17⋅4			
Obese	26	11⋅9			
Waist (cm)			54⋅0–107⋅3	71⋅6	10⋅5
<88	216	91⋅5			
≥88	20	8⋅5			
Total	236	100			
Hb level (mg/l)			86–161	129⋅5	13
≥120 (no anaemia)	195	81⋅9			
<120 (anaemic)	43	18⋅1			

Table 3 shows the sociodemographic characteristics of the study participants by weight status and Hb level. Females aged 20 years or younger were more likely to be underweight (20⋅9 %), while females older than 20 years were more likely to be overweight (20⋅8 %). In addition, anaemia was more prevalent among female students aged >20 years old (23 *v.* 14⋅5 %), with no significant difference (*P* *=* 0⋅066).

**Table 3.** Sociodemographic characteristics by weight status and Hb level (Percentages of subjects)

Characteristic	Weight status (%)				P*	Hb level (%)		P*
	Underweight (*n* 37)	Normal weight (*n* 117)	Overweight (*n* 38)	Obese (*n* 26)		Anaemia (*n* 43)	Normal (*n* 195)	
Age								
≤20 years	20⋅9	53⋅2	14⋅4	11⋅5	0⋅463	14⋅5	85⋅5	0⋅066
>20 years	14⋅9	52⋅5	20⋅8	11⋅9		23⋅0	77⋅0	
Academic level								
1st year	30⋅0	40⋅0	17⋅5	12⋅5	0⋅736	20⋅5	79⋅5	0⋅233
2nd year	17⋅0	53⋅2	14⋅9	14⋅9		8⋅5	91⋅5	
3rd year	15⋅7	57⋅1	17⋅1	10⋅0		23⋅2	76⋅8	
4th year	15⋅7	55⋅4	18⋅1	10⋅8		18⋅1	81⋅9	
Economic status perception								
High	16⋅8	64⋅5	14⋅4	4⋅3	0⋅324	11⋅9	88⋅1	0⋅168
Moderate	17⋅9	51⋅3	17⋅4	13⋅3		19⋅7	80⋅3	
Marital status								
Single	18⋅5	51⋅7	16⋅6	13⋅3	0⋅432	16⋅7	83⋅3	0⋅492
Engaged	16⋅7	55⋅6	27⋅8	0⋅0		27⋅8	72⋅2	
Married	20⋅0	70⋅0	10⋅0	0⋅0		20⋅0	80⋅0	

* χ^2^ Test.

The prevalence of underweight was found to be the highest among female students in their 1st year of study (30 %). No association was found between overweight, obesity and year of study (Table 3). Overweight, obesity and anaemia were more prevalent among female students who perceived that their families have moderate economic status (17⋅4, 13⋅3 and 19⋅7 %, respectively) than those who perceived that their families have high economic status (14⋅4, 4⋅3 and 11⋅9 %, respectively) (Table 3).

Table 4 shows that abdominal obesity (WC ≥88 cm) was more prevalent among obese students than among overweight and normal-weight students (*P* < 0⋅001). The percentage of total body fat measured by the Tanita machine was the highest among obese and overweight students, while only 2⋅9 % among normal-weight students (*P* < 0⋅001). Moreover, the percentage of total body fat was found to be the highest (38⋅9 %) among students with anaemia (*P* < 0⋅05) (Table 4).

**Table 4.** Associations of weight status and Hb level with waist circumference, percentage of total body fat and selected lifestyle characteristics (Numbers of subjects and percentages)

Characteristic	Total		Weight status (%)					Hb level (%)		
	*n*	%	Under	Norm	Over	Obese	P*	Anaemia	Normal	P*
Waist (cm)										
<88	216	91⋅5	100⋅0	99⋅2	92⋅7	40⋅7	*P* < 0⋅001	90⋅5	91⋅4	0⋅507
≥88	20	8⋅5	0⋅0	0⋅8	7⋅3	59⋅3		9⋅5	8⋅6	
Total	236	100	100	100	100	100		100	100	
Total body fat										
Athletic (8–15 %)	12	5⋅9	28⋅2	1⋅0	0⋅0	0⋅0		8⋅3	5⋅5	
Good (16–23 %)	39	19⋅2	61⋅5	14⋅4	0⋅0	0⋅0	*P* < 0⋅001	25⋅0	18⋅3	0⋅030
Acceptable (24–30 %)	63	31⋅1	10⋅3	55⋅8	3⋅0	0⋅0		25⋅0	32⋅9	
Overweight (31–36 %)	38	18⋅7	0⋅0	26⋅0	30⋅3	3⋅7		2⋅8	21⋅3	
Obese (≥37 %)	51	25⋅1	0⋅0	2⋅9	66⋅7	96⋅3		38⋅9	22⋅0	
Total	203	100	100	100	100	100		100	100	
Sleeping (h/d)										
Less than 7	91	37	37⋅2	33⋅3	36⋅6	46⋅4	0⋅669	43⋅9	35⋅4	0⋅560
7–8	123	50	48⋅8	55⋅3	43⋅9	42⋅9		43⋅9	52⋅6	
More than 8	32	13	14⋅0	11⋅4	19⋅5	10⋅7		12⋅2	12⋅0	
Total	246	100	100	100	100	100		100	100	
Exercise ≥60 min										
Yes	140	56	38⋅6	56⋅7	67⋅5	60⋅7	0⋅050	44⋅2	59⋅3	0⋅050
No	110	44	61⋅4	43⋅3	32⋅5	39⋅3		55⋅8	40⋅7	
Total	250	100	100	100	100	100		100	100	
Meal pattern										
Always regular	71	28⋅3	22⋅7	34⋅6	22⋅0	10⋅7	0⋅039	23⋅3	28⋅7	0⋅301
Irregular	180	71⋅7	77⋅3	65⋅4	78⋅0	89⋅3		76⋅7	71⋅3	
Total	251	100	100	100	100	100		100	100	
Breakfast pattern										
Regular	105	41⋅8	29⋅5	46⋅5	43⋅9	39⋅3	0⋅439	41⋅9	43⋅1	0⋅942
Irregular	129	51⋅4	63⋅6	45⋅7	53⋅7	53⋅6		51⋅2	51⋅3	
I don't eat breakfast	17	6⋅8	6⋅8	7⋅9	2⋅4	7⋅1		7⋅0	5⋅6	
Total	251	100	100	100	100	100		100	100	
Dieting to lose weight										
Yes	61	24⋅4	2⋅3	17⋅5	43⋅9	53⋅6	*P* < 0⋅001	18⋅6	25⋅3	0⋅237
No	189	75⋅6	97⋅7	82⋅5	56⋅1	46⋅4		81⋅4	74⋅7	
Total	250	100	100	100	100	100		100	100	
Prefer junk food										
Yes	150	59⋅8	59⋅1	58⋅3	63⋅4	71⋅4	0⋅606	72⋅1	57⋅4	0⋅053
No	101	40⋅2	40⋅9	41⋅7	36⋅6	28⋅6		27⋅9	42⋅6	
Total	251	100	100	100	100	100		100	100	

Under, underweight; Norm, normal weight; Over, overweight.

* χ^2^ Test.

Obese and overweight students reported that they exercise (at least 60 min/d) more than normal-weight and underweight students (*P* = 0⋅05). In addition, students with anaemia reported less exercise (60 min/d) than students without anaemia (*P* = 0⋅05). More than two-thirds of the students (71⋅7 %) reported that they follow irregular meals pattern. Obese and overweight students reported having irregular meals patterns more than normal-weight students (*P* < 0⋅05) (Table 4).

More than half of the obese (53⋅6 %) and 43⋅9 % of the overweight students reported that they were currently on a diet to lose weight, while only 17⋅5 % of the normal-weight and 2⋅3 % of the underweight students reported that they were dieting (*P* < 0⋅001). In addition, about 60 % of the participants reported that they prefer junk food and the highest percentage was found among obese students (71⋅4 %), followed by overweight students (63⋅4 %). More than two-thirds (72⋅1 %) of the anaemic students stated junk food preference compared with 57⋅4 % of the non-anaemic (*P* = 0⋅053) (Table 4).

## Discussion

The present study aimed to assess the prevalence of overweight, obesity and anaemia and their associations with sociodemographic characteristics, lifestyle, total body fat and abdominal obesity among female university students in Dubai. Based on our current knowledge, this is the first study conducted among female university students in Dubai covering this topic. A systematic random sampling approach was used to ensure that the selected students were representative of all the university's female students and findings of the present study can be generalised to all UAE and Gulf Cooperation Council university's female students.

Both obesity and anaemia are worldwide epidemics affecting vulnerable populations^(19)^. In the present study, 17⋅4 % of the participants were found to be overweight, 11⋅9 % were obese and 8⋅5 % had abdominal obesity following the WHO classification^(2)^. Abdominal obesity prevalence has not been reported before among female university students in Dubai. Abdominal obesity is associated with increased risk of type 2 diabetes, CVD, sleep apnoea and premature death^(20)^. The prevalence of obesity found in the present study is close to the WHO's estimates and highlights the importance of obesity as a public health problem^(21)^.

About one-fifth (18⋅1 %) of the study participants were anaemic based on the WHO cut-off values^(22)^. Hb concentration can indicate the severity of anaemia^(18)^. The present study found that a high level of total body fat was associated with anaemia (*P* = 0⋅030). Also, low levels of exercise and preferences to eat junk food were found to be associated with anaemia. This might be explained by the fact that poor dietary choices – which has been reported in the present study as high preference for ‘junk food’ − are generally deficient in essential nutrients especially Fe or have substances that reduce Fe absorption. The students with anaemia reported a high tendency to engage in dieting behaviour at the time of the study, which can explain our results if they were following fad diets that are based on eliminating major food groups. Also, students with anaemia have an increased percentage of total body fat which can make exercise harder in addition to their anaemia.

Obesity may stimulate anaemia by inhibiting dietary Fe uptake from the duodenum^(23)^. Several analyses have demonstrated an association between lower serum Fe concentrations with higher BMI, particularly in women^(21)^. The present study found significant associations between anaemia and total body fat. Obese and underweight students were found to be two times more likely to have anaemia compared with normal-weight students. This might be due to the fact that anaemia is one of the most common nutritional deficiency disorders^(24,25)^.

The nature of the cross-sectional study design poses a challenge in inferring causal relationships, especially when examining temporality. Whether obesity has led to anaemia and lack of exercise or poor diet choices and lack of activity led to obesity and anaemia cannot be determined from the results of the present study. While the present study investigated dieting behaviours, it did not ask for a complete food recall which could have led to different interpretations of what can ‘junk food’ mean.

### Conclusions

Overweight, obesity and anaemia are prevalent among female university students in Dubai. The present study found associations between anaemia, lack of physical activity and percentages of total body fat among young female adults. This may be related to unbalanced diets and preferences for junk food as reported in the present study. Health promotion and nutritional education programmes are needed to reduce the prevalence of anaemia, overweight and obesity among female university students.

Further studies are required to investigate the detailed dietary habits of overweight and obese young adult females with anaemia.
